# Molecular and Cellular Mechanisms of Aging and Age-related Disorders

**DOI:** 10.3390/ijms19072049

**Published:** 2018-07-14

**Authors:** Vladimir I. Titorenko

**Affiliations:** Department of Biology, Concordia University, Montreal, 7141 Sherbrooke Street, West, QC H4B1R6, Canada; vladimir.titorenko@concordia.ca; Tel.: +1-514-848-2424

Aging of unicellular and multicellular eukaryotic organisms is a convoluted biological phenomenon, which is manifested as an age-related functional decline caused by a progressive dysregulation of certain cellular and organismal processes [1]. Many chronic diseases are associated with human aging. These aging-associated diseases include cardiovascular diseases, chronic obstructive pulmonary disease, chronic kidney disease, diabetes, osteoarthritis, osteoporosis, sarcopenia, stroke, neurodegenerative diseases (including Parkinson’s, Alzheimer’s and Huntington’s diseases), and many forms of cancer [2,3]. Studies in yeast, roundworms, fruit flies, fishes, mice, primates and humans have provided evidence that the major aspects and basic mechanisms of aging and aging-associated pathology are conserved across phyla [4,5]. The focus of this *International Journal of Molecular Sciences* Special Issue is on molecular and cellular mechanisms of aging and age-related disorders. Eight original research and review articles of the Issue provide important insights into how various genetic, dietary and pharmacological interventions can affect certain longevity-defining cellular processes to delay aging and postpone the onset of age-related pathologies in evolutionarily diverse organisms. 

Krajewska-Włodarczyk et al. review mechanisms underlying the development of aging-associated changes in human chondrocytes, such as telomere shortening, a build-up of reactive oxygen species, an increase in the extent of oxidative damage to cellular macromolecules, a rise in the concentrations and secretion of inflammatory cytokines and matrix metalloproteinases, a decline in the responsiveness to growth factors, a delay in morphogenesis and maturation, and an accumulation of proteoglycan fragmentation products and advanced glycation end-products [6]. The authors discuss how these aging-associated changes in human chondrocytes can impair the mechanical properties of articular cartilage and how they can lead to the development of osteoarthritis. They also explore several strategies and approaches for the development of therapeutic interventions that can be used to delay the aging-associated impairment of articular cartilage and/or to improve the quality of aged articular cartilage.

Zöller et al. demonstrate that aging in mice correlates with a specific remodeling of gene expression in cortical microglia, the resident immune cells of the central nervous system [7]. This remodeling consists in activated expression of genes implicated in innate immune responses, cholesterol/steroid metabolism, purine nucleotide biosynthesis, and the M1- and M2-like types of microglia activation. The authors propose that such aging-associated remodeling of gene expression in cortical microglia (1) may allow these cells to acquire specific immunoregulatory and/or anti-inflammatory properties needed to sustain neuron survival, and (2) may also promote neurodegeneration by eliciting the M1-like type of microglia activation in some regions of the aged central nervous system.

Garcia-Contreras et al. provide evidence that aged female Iberian pigs exhibiting clinical symptoms of aging-associated sarcopenia and fed a standard diet or an obesogenic diet represent an adequate preclinical model for studying aging-associated sarcopenia and sarcopenic obesity (respectively) in humans [8]. They demonstrate that, like in human sarcopenia, aging-associated sarcopenia in these pigs coincides with dyslipidemia, insulin resistance, lipotoxicity, and morphological changes in the liver characteristic of human early-stage nonalcoholic fatty liver disease and nonalcoholic steatohepatitis. They also show that, akin to human sarcopenic obesity, the aged female pigs fed an obesogenic diet with saturated fat exhibit enlarged subcutaneous and visceral fats, substantial changes in the plasma lipids profile, a rise in total cholesterol, an increase in low-density lipoproteins cholesterol, low concentrations of high-density lipoproteins cholesterol, compromised glucose regulation, hypertriglyceridemia, systemic oxidative stress and steatosis in non-adipose tissues, insulin resistance, elevated fatty acid desaturation index, and activated stearoyl-CoA desaturase 1.

Mohammad et al. analyze and critically evaluate findings suggesting that the spatiotemporal dynamics of age-related changes in the intracellular and extracellular concentrations of some key metabolic intermediates may regulate longevity of chronologically aging budding yeast [9]. These metabolites include reduced nicotinamide adenine dinucleotide phosphate, glycerol, trehalose, hydrogen peroxide, amino acids, sphingolipids, spermidine, hydrogen sulfide, acetic acid, ethanol, free fatty acids and diacylglycerol. Their analysis reveals that these key metabolites perhaps act as second messengers that define the rate of yeast chronological aging. The authors discuss (1) how changes in the nutrient, energy, stress and proliferation status of a yeast cell alter the abundance and cellular location of each of these second messengers of aging at different stages of the chronological aging process; (2) how the alterations in the concentrations of these second messengers of aging influence cell functionality and how they affect the chance of cell survival throughout chronological lifespan; (3) what are the mechanisms through which the spatiotemporal dynamics of changes in the concentrations of these second messengers of aging define yeast chronological lifespan, and (4) what are the common properties of these second messengers of aging and second messengers of signal transduction.

Zhang et al. report that intermittent fasting, a dietary intervention that extends longevity and delays the onset of age-related neurodegeneration in *Drosophila*, exhibits the following effects on male flies: (1) it delays aging-associated changes in transcriptomes of neural and skeletal muscle tissues; (2) it slows down aging-associated changes in whole-body metabolomes, and (3) it suppresses an aging-associated increase in nighttime activity [10]. The authors demonstrate that the effects of intermittent fasting on such aging-associated changes in whole-body metabolomes and adult olfactory-based behaviors correlate with aging-associated alterations in transcription of genes that have been implicated in these metabolic processes and behavioral patterns. They also show that an aging-associated rise in protein aggregation that occurs in the neural tissue of male files exhibits a correlation with transcriptional profiles of genes involved in cellular proteostasis. Based on these findings, Zhang et al. conclude that both aging and aging delay by a mild diet intervention define longevity of male flies by altering transcription of a distinct set of longevity-defining genes in a tissue-specific manner. They propose that these changes in transcription of longevity-defining genes occur in early adulthood, perhaps due to the epigenetic plasticity of chromatin in different tissues, to define the rates of aging of individual tissues and the entire organism. They also infer that even such mild dietary regimen as intermittent fasting, which in humans would likely have less negative side effects and amenability issues as compared to caloric restriction, can have significant extending effects on both lifespan and healthspan of male flies.

Wang et al. show that the electronegative low-density lipoprotein L5, an atherogenic risk factor, is also a neurotoxic factor that (1) kills cultured rat PC12 cells in a concentration-dependent manner, and (2) if used in sublethal concentrations, inhibits neurite outgrowth of these neuron-like cells pre-treated with nerve growth factor [11]. They provide evidence that both these neurotoxic effects of L5 are mediated by the lectin-like oxidized low-density lipoprotein receptor-1 (LOX-1). The authors propose that, because the atherogenic risk factor L5 is neurotoxic in vitro, it may contribute to neurodegenerative disorders in vivo by targeting LOX-1 to generate a neurotoxic stress. They also suggest that, because high levels of circulating low-density lipoproteins have been implicated in the pathophysiology of such neurodegenerative disorder as Alzheimer’s disease-type dementia, the severity of both cognitive impairment and Alzheimer’s disease may be weakened by inhibiting electronegative modification of circulating low-density lipoproteins to decrease their electronegativity in plasma. 

Harkness provides deep insights into mechanisms through which the yeast Anaphase Promoting Complex (APC), an evolutionarily conserved large ubiquitin-protein ligase, extends yeast replicative lifespan [12]. The author offers a thought-provoking discussion of the following properties of the APC as a longevity assurance factor in replicatively aging yeast: (1) the APC is dynamically integrated into a signaling network that also assimilates several nutrient-sensing and stress-response signaling pathways known to regulate longevity of replicatively aging yeast; (2) the APC is involved in controlling cell cycle progression, promoting repair of DNA damage incurred during chromosome segregation and maintaining genomic stability; all these processes also define longevity of replicatively aging yeast, and (3) the APC coordinates the multidirectional information flow along the signaling network that is modulated in response to changes in the nutrient, stress and/or genome stability status of a replicatively aging yeast cell. Because different forms of cancers are believed to be aging-associated diseases, the author also discusses strategies that can be used for the development of anti-tumor therapeutic interventions specifically targeting the APC in cancer cells.

Palomino-Alonso et al. use a combination of the RNA microarray-based transcriptomic and mass spectrometry-based proteomic analyses to identify genes and proteins whose abundance is altered in the olfactory bulb of aged transgenic Tg2576 mice, an Alzheimer’s disease model known to overexpress the Swedish mutated form of human amyloid precursor protein [13]. They find 107 protein-coding genes that are up- or down-regulated in comparison with the olfactory bulb of age-matched wild-type mice; these genes encode proteins implicated in cell response to cyclic adenosine monophosphate and in different steps of the olfactory transduction signaling. They also identify 25 proteins that are differentially regulated in aged transgenic Tg2576 mice; these proteins are known to be involved in membrane biogenesis, protein trafficking and negative control of neuron differentiation. Their bioinformatic pathway analysis of the regulatory and metabolic networks that are dysregulated in aged transgenic Tg2576 mice, immunoblotting analysis of the abundance of proteins involved in survival and apoptotic pathways, functional interactome analysis by network-driven proteogenomics, immunoblotting analysis of the cyclic AMP-responsive element-binding protein 1 and protein products of the genes whose transcription is regulated by this transcription factor, and phosphorylation status analysis of the olfactory protein complex functionality suggest that the impairment of olfaction in aged Tg2576 Alzheimer’s disease mouse model is likely due to specific changes in protein–protein interactions, post-translational protein modifications, impaired intracellular protein trafficking and/or compromised cellular proteostasis.
